# Rate of Force Development Is Related to Maximal Force and Sit-to-Stand Performance in Men With Stages 3b and 4 Chronic Kidney Disease

**DOI:** 10.3389/fresc.2021.734705

**Published:** 2021-09-28

**Authors:** Jared M. Gollie, Michael O. Harris-Love, Samir S. Patel, Nawar M. Shara, Marc R. Blackman

**Affiliations:** 1 Skeletal Muscle Laboratory, Research Service, Washington, DC, United States,; 2 George Washington University, Health, Human Function, and Rehabilitation Sciences, Washington, DC, United States,; 3 George Mason University, Rehabilitation Science, Fairfax, VA, United States,; 4 Department of Physical Medicine and Rehabilitation, University of Colorado Anschutz Medical Campus, Aurora, CO, United States,; 5 Muscle Morphology, Mechanics and Performance Laboratory, Eastern Colorado VA Geriatric Research Education and Clinical Center, Aurora, CO, United States,; 6 Renal Service, Washington DC VAMC, Washington, DC, United States,; 7 Department of Medicine, George Washington University, Washington, DC, United States,; 8 Department of Biostatistics and Biomedical Informatics, MedStar Health Research Institute, Washington, DC, United States,; 9 Research Service, Washington DC VAMC, Washington, DC, United States,; 10 Departments of Medicine and Rehabilitation Medicine, Georgetown University, Washington, DC, United States

**Keywords:** chronic kidney disease, maximal voluntary force, strength, muscle quality, physical function

## Abstract

**Introduction:**

The primary aims of the present study were to assess the relationships of early (0–50 ms) and late (100–200 ms) knee extensor rate of force development (RFD) with maximal voluntary force (MVF) and sit-to-stand (STS) performance in participants with chronic kidney disease (CKD) not requiring dialysis.

**Methods:**

Thirteen men with CKD (eGFR = 35.17 ±.5 ml/min per 1.73 m^2^, age = 70.56 ±.4 years) and 12 non-CKD men (REF) (eGFR = 80.31 ± 4.8 ml/min per 1.73 m^2^, age = 70.22 ±.9 years) performed maximal voluntary isometric contractions to determine MVF and RFD of the knee extensors. RFD was measured at time intervals 0–50 ms (RFD_0–50_) and 100–200 ms (RFD_100–200_). STS was measured as the time to complete five repetitions. Measures of rectus femoris grayscale (RF GSL) and muscle thickness (RF MT) were obtained via ultrasonography in the CKD group only. Standardized mean differences (SMD) were used to examine differences between groups. Bivariate relationships were assessed by Pearson’s product moment correlation.

**Results:**

Knee extensor MVF adjusted for body weight (CKD=17.14 ±.1 N·kg^0.67^, REF=21.55 ±.3 N·kg^0.67^, SMD = 0.79) and STS time (CKD = 15.93 ±.4 s, REF = 12.23 ±.7 s, SMD = 1.03) were lower in the CKD group than the REF group. Absolute RFD_100–200_ was significantly directly related to adjusted MVF in CKD (*r* = 0.56, *p* = 0.049) and REF (*r* = 0.70, *p* = 0.012), respectively. STS time was significantly inversely related to absolute (*r* = −0.75, *p* = 0.008) and relative RFD_0–50_ (*r* = −0.65, *p* = 0.030) in CKD but not REF (*r* = 0.08, *p* = 0.797; *r* = 0.004, *p* = 0.991). Significant inverse relationships between RF GSL adjusted for adipose tissue thickness and absolute RFD_100–200_ (*r* =−0.59, *p* = 0.042) in CKD were observed.

**Conclusion:**

The results of the current study highlight the declines in strength and physical function that occur in older men with CKD stages 3b and 4 not requiring dialysis. Moreover, early RFD was associated with STS time in CKD while late RFD was associated MVF in both CKD and REF.

**Clinical Trial Registration:**

ClinicalTrials.gov, identifier: NCT03160326 and NCT02277236.

## INTRODUCTION

Reductions in neuromuscular capacity with aging are accelerated in the presence of chronic kidney disease (CKD) (1–4). Metabolic derangements affecting neuromuscular capacity, including anemia, hyperparathyroidism, metabolic acidosis, sodium retention, and hyperkalemia increase in prevalence and severity with CKD progression, usually becoming clinically apparent with an estimated glomerular filtration rate (eGFR) of <45 ml/min per 1.73 m^2^ (5). The accumulation of uremic toxins resulting from declines in kidney function are reported to contribute to both neuropathy and myopathy (6–9). Neuromuscular complications are further compounded by age, comorbidities, and high levels of physical inactivity in the CKD population (3, 10–12).

Peripheral neuropathy and altered nerve excitability are common clinical manifestations of CKD and end-stage kidney disease (ESKD) (8, 13–15). Similarly, skeletal muscle wasting and mitochondrial dysfunction are also known to occur in those with CKD (16, 17). Accordingly, both neural and muscle morphological alterations are potential factors contributing to muscle weakness. The loss in neuromuscular force is reported to occur more rapidly than changes in muscle cross-sectional area in patients with CKD predialysis and ESKD (18, 19). Moreover, changes in muscle cross-sectional area have been found to be poorly associated with changes in functional outcomes (18). Therefore, alterations in neuromuscular force characteristics, in addition to loss of skeletal muscle mass, may explain declines in physical function in CKD (20).

Rate of force development (RFD) describes the rise in force per unit of time following the onset of muscle contraction and is considered a critical contributor to the performance of functional activities (21–26). The underlying mechanisms determining RFD include a combination of neural and muscular factors (27, 28). More specifically, neural factors are suggested to predominantly influence the initial rise in force following the onset of muscle contraction (<100 ms) while skeletal muscle factors become more prominent during the later stages of RFD (>100 ms) (29–32). Decreases in muscle activation, maximal force capacity, and muscle quality, and alterations in muscle architecture have been reported to contribute to reductions in both early and late time intervals of RFD in older adults (24, 33–37). However, the effects of neuromuscular factors on early and late RFD and the potential implications for physical functioning in CKD is less understood.

To our knowledge, there are no prior reports describing the relationship between RFD and physical function in patients with moderate-to-severe CKD. Therefore, the primary aims of the present study were to assess the relationships of early (0–50 ms) and late (100–200 ms) knee extensor RFD with maximal voluntary force (MVF) and sit-to-stand (STS) performance in patients with stages 3b and 4 CKD not requiring dialysis.

## MATERIALS AND METHODS

### Participants and Ethical Approval

Community-dwelling male veterans were screened and referred for potential enrollment by the Renal Clinic staff at the Veterans Affairs Medical Center in Washington D.C. (DC VAMC). The study was approved by the DC VAMC Institutional Review Board (IRB) and Research and Development (R&D) Committee. Inclusion criteria for study enrollment required participants to be ambulatory, with or without use of an assistive device, aged 18–85 years, and diagnosed with CKD stage 3b or 4 (i.e., eGFR >15 to <45 ml/min per 1.73 m^2^) (10). Exclusion criteria included a history of acute kidney injury, any uncontrolled cardiovascular or musculoskeletal problems, or having a pacemaker or implantable cardioverter defibrillator. Men similar in age without clinical evidence of CKD (i.e., eGFR >60 ml/min per 1.73 m^2^), who were recruited as part of a separate study in our laboratory using identical strength and functional testing procedures, served as a reference group (REF). All CKD and REF study participants voluntarily provided written informed consent using a DC VAMC IRB and R&D approved form prior to study participation.

### Protocol

We employed an exploratory, prospective, case-referent study design comparing skeletal muscle force characteristics and physical function in male veterans with stages 3b and 4 CKD not on dialysis (*n* = 13) to male veterans without CKD (*n* = 12). Study participants included a convenience sample of middle-aged and older men seen at the DC VAMC. Data were collected by trained staff in the Renal Clinic, and Physical Medicine and Rehabilitation and Research Service groups. Each participant reported to the Muscle Morphology, Mechanics, and Performance Laboratory (3MAP Lab) at the DC VAMC to complete neuromuscular strength and physical function assessments lasting a total of 60–90 min. Participants were asked to refrain from any vigorous activity for at least 24 h prior to testing. Functional performance was determined using the STS task and knee extensors force characteristics of the dominant leg were determined using maximal voluntary isometric contractions. Diagnostic ultrasound examinations of the dominant rectus femoris muscle was performed in all patients with CKD. Functional assessments were completed prior to strength testing, and diagnostic ultrasound imaging was completed prior to all physical performance tasks.

### Sit-to-Stand (STS) Test

The STS task has been proposed as a measure of lower extremity strength and power (38–41). To complete the STS, participants were asked to perform five repetitions of the STS task as quickly, and safely, as possible. Participants began by sitting quietly on a seat (height 43 cm) with their hands folded across their chest. If the participant was able to complete one STS repetition successfully without using his arms, he was provided the following standardized instructions for completing the test: “Please stand up straight as quickly as you can five times, without stopping in between. After standing up each time, sit down and then stand up again while keeping your arms folded across your chest”. Performance in the STS was determined by the time taken to successfully complete all five repetitions. The test was ended if the participant became tired or short of breath, used his arms, was unable to complete the five repetitions within 1 min, or if the test administrator became concerned about the participant’s safety.

### Maximal Voluntary Force (MVF) Assessment

Knee extensor MVF of the dominant leg was assessed using a portable fixed dynamometer (FGV-200XY, NIDEC instruments, Itasca, IL) (42). The dynamometer was securely mounted and anchored behind the participant. Participants were placed in the seated position on the edge of an adjustable examination table with their hips and knees at 90° of flexion. A strap attached to the dynamometer was secured around the ankle of the participant’s dominant leg. Participants were allowed to use their hands for stability but were asked to refrain from grasping onto the table. A familiarization session was provided before data collection to orient each participant to the testing procedures. Participants were instructed to extend or kick their leg as “hard and fast” as possible immediately following the test administrator’s command, and to contract and sustain the contraction for 5-s while being provided strong verbal encouragement. A rest period of ~1-min was provided between repetitions. Pre-contraction tension and countermovement were removed by test administrators and the dynamometer was zeroed just before initiation of contraction.

The real-time force applied to the dynamometer was displayed online on a computer monitor. Force data were sampled at 100 Hz and digitally stored to be analyzed online. No more than six maximal voluntary isometric contractions were completed during strength assessments. Absolute MVF was determined as the average of the instantaneous highest force of 2–3 attempts which fell within 10% of each other. Absolute MVF was also adjusted for body weight and calculated as absolute MVF (N) divided by body weight (kg) to two-thirds power (i.e., adjusted MVF = absolute MVF [N]/body weight [kg]^0.67^) (43).

Absolute RFD was calculated as the change in force by the change in time over the first 300 ms of the knee extensor isometric contraction. Absolute RFD was then analyzed at time intervals 0–50 ms (RFD_0–50_) and 100–200 ms (RFD_100–200_) representing early and late phases of RFD. Several steps were taken to increase confidence in the RFD measures. First, pre-tension prior to the onset of muscle contraction was eliminated. Second, caution was taken to ensure no counter-movements occurred before the start of the test. Finally, only trials exhibiting a rapid rise in the force trace were used for analysis. The average of 2–3 trials were used to determine RFD for each participant. The onset of contraction was systematically determined as the last trough before a continuous rise in the filtered force signaling above 4.45 N using visual identification (27). Relative RFD was expressed as a percentage of MVF (27, 43).

### Diagnostic Ultrasound Estimates

Estimates of rectus femoris (RF) muscle quality were assessed as levels of echogenicity and expressed as grayscale levels (GSL) (range, 0–255) (44–46). Estimates of RF muscle thickness (MT) were determined from longitudinal view images. Images were obtained by a single clinical investigator with more than 10 years of quantitative ultrasound experience using B-mode diagnostic ultrasound with a 5–18 MHz linear array transducer (Nobulus, Hitachi Aloka Medical, Parsippany, NJ). Manufacturer default settings for time gain compensation and near field/far field gain were applied for all musculoskeletal scans. The field of view was adjusted for each participant to optimally capture the region of interest of the RF on the dominant side. Standardized procedures were followed for the identification of the scanning site, as previously described (45, 47).

Diagnostic ultrasound estimates of RF GSL and RF MT were completed using ImageJ (version 1.48; National Institutes of Health, Bethesda, MD, USA). Ultrasound images were obtained and measured 3 times within the fascial borders of the muscle of interest. The region of interest was defined as the area within the superior and inferior fascial borders and the lateral borders of the RF. In instances when a portion of a fascial border was poorly visualized, the examiner used the trajectory of the visible fascial border to complete the region of interest selection (45). RF GSL estimates were adjusted for subcutaneous fat (i.e., adjusted RF GSL = uncorrected GSL + (subcutaneous fat thickness [cm] × 40.5278)) while RF MT estimates were adjusted for body weight (i.e., RF MT = muscle thickness [mm]/body weight [kg]) (48, 49).

### Statistical Analysis

All data met the assumptions of normality based on the Kolmogorov-Smirnov test. The exploratory nature of the study precluded the use of a formal power calculation for estimating sample size. Therefore, standardized mean differences (SMD) were used to examine potential differences between CKD and REF groups in demographic data, force characteristics, and STS performance (50, 51). SMD provide an assessment of the amount of non-overlap in the distributions of two (or more) statistics (means, proportions) regardless of the number of participants in a study. SMD of > 0.2 (or < −0.2) indicates about 15% non-overlap in distributions while a difference of 1.0 would indicate more than 50% non-overlap. Pearson’s product moment bivariate correlation coefficients (r) were calculated to determine the associations between force characteristics, STS performance, and diagnostic ultrasound measures. The magnitudes of the SMD and correlation coefficients were described as 0.2, 0.5, and 0.8 and interpreted as small, medium, and large, respectively (52). Data are presented as mean values ± standard deviation (SD) unless otherwise noted and statistical significance was set at *p* ≤ 0.05. Statistical analyses were performed using SPSS version 26 (SPSS Inc., Chicago, IL).

## RESULTS

### Demographics

Participant characteristics are presented in Table 1. Adults with CKD were classified as exhibiting moderate-to-severe kidney insufficiency based on mean eGFR values, with nine (69.2%) participants classified with stage 3b and four (30.8%) classified with stage 4. Nine CKD patients (69%) and four REF participants (33%) had a clinical diagnosis of diabetes mellitus, whereas 12 CKD patients (92%) and 11 REF participants (92%) were hypertensive. Using BMI criteria, five CKD patients (38.5%) were classified as overweight, and eight (61.5%) were classified as obese (53). In the REF group, four (33.3%) were classified as overweight, and eight (66.6%) as obese.

### Force Characteristics and STS Performance

Two CKD participants were unable to perform a single STS repetition and therefore were excluded from STS analyses. Functional status differed between groups, as evidenced by the large effect in STS time between CKD and REF groups. The difference of 3.7 seconds in STS time between groups surpassed the calculated minimal detectable change (MDC) of 1.2 s previously reported in non-dialysis patients (54). Medium-to-large effects were observed in MVF (SMD = 0.53) and adjusted MVF (SMD = 0.79), with absolute and adjusted strength being lower in the CKD group as compared to the REF group. Medium effects for relative RFD_0–50_ (SMD = 0.54) and large effects for relative RFD_100–200_ (SMD = 0.98) were observed between groups with relative RFD being greater in the CKD group compared to REF group.

### Bivariate Relationships Between Absolute RFD and MVF Measures

**FIGURE 1 |:**
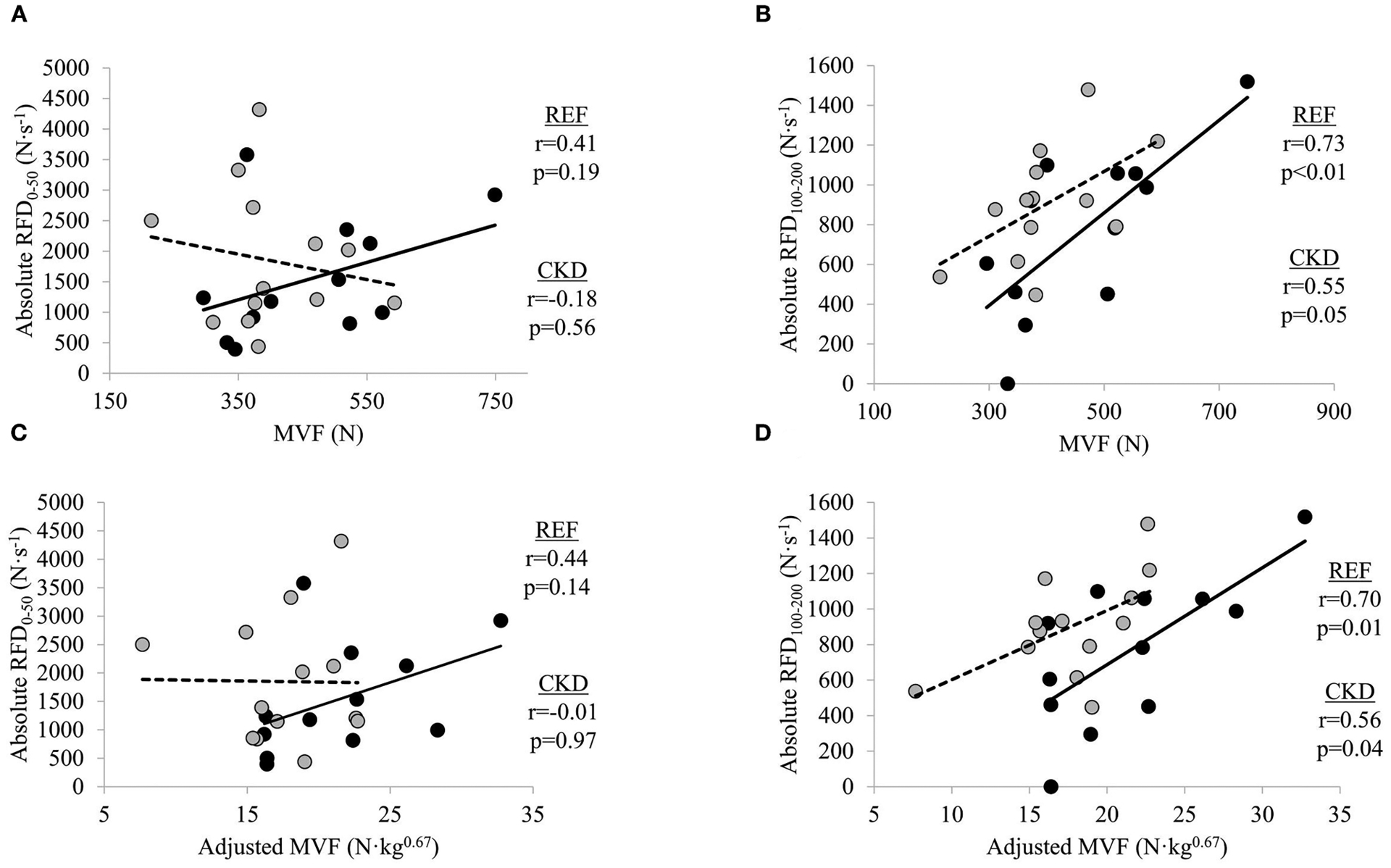
Relationships between absolute rate of force development (RFD) and maximal voluntary force (MVF) at time intervals (**A**) 0–50 ms and (**B**) 100–200 ms, and absolute RFD and adjusted MVF at time intervals (**C**) 0–50 ms and (**D**) 100–200 ms. Gray circles and dashed lines represent patients with chronic kidney disease stages (CKD) 3b and 4 not on dialysis (CKD) while solid circles and lines represent reference participants (REF). *N*, newtons; *N*·*s*^−1^, newtons per second. Significance level set at *p* ≤ 0.05. *N*·*kg*^0.67^, newtons per kilogram to the two-thirds power.

In adults with CKD, negligible-to-weak non-significant inverse relationships were seen between absolute RFD_0–50_ and MVF (*r* = −0.18, *p* = 0.562) and absolute RFD_0–50_ and adjusted MVF (*r* = −0.01, *p* = 0.973) while absolute RFD_100–200_ was moderately directly related to MVF (*r* = 0.55, *p* = 0.054) and significantly moderately directly related adjusted MVF (*r* = 0.56, *p* = 0.049) (Figure 1). In the REF group, non-significant small-to-moderate direct relationships were observed between absolute RFD_0–50_ and MVF (*r* = 0.41, *p* = 0.191) and absolute RFD_0–50_ and adjusted MVF (*r* = 0.44, *p* = 0.149) whereas absolute RFD_100–200_ was significantly moderately directly related to MVF (*r* = 0.73, *p* = 0.007) and adjusted MVF (*r* = 0.70, *p* = 0.012).

### Bivariate Relationships Between RFD Measures and STS Performance

**FIGURE 2 |:**
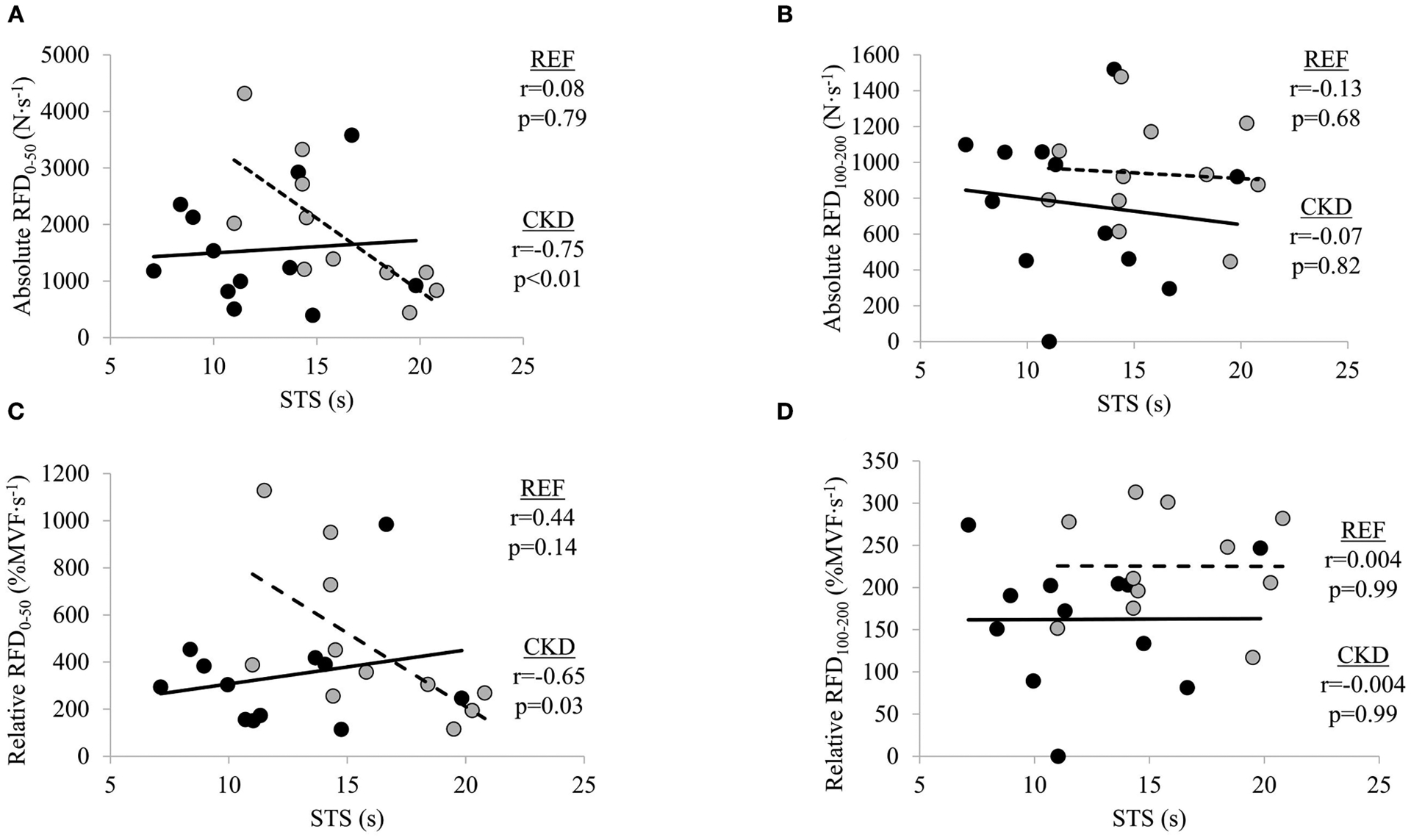
Relationships between absolute rate of force development (RFD) and sit-to-stand (STS) at time intervals (**A**) 0–50 ms and (**B**) 100–200 ms, and relative RFD and STS at time intervals, (**C**) 0–50 ms and (**D**) 100–200 ms. Gray circles and dashed lines represent patients with chronic kidney disease stages (CKD) 3b and 4 not on dialysis (CKD) while solid circles and lines represent reference participants (REF). *N*·*s*^−1^, Newtons per second; %*MVF*·*s*^−1^, percent maximal voluntary force per second; *s*, seconds. Significance level set at *p* ≤ 0.05.

There were significant moderate inverse relationships between STS time and absolute RFD_0–50_ (*r* = −0.75; *p* = 0.008) and relative RFD_0–50_ (*r* = −0.65; *p* = 0.030) in the CKD group but not absolute RFD_100–200_ (*r* = −0.07; *p* = 0.829) or relative RFD_100–200_ (*r* = −0.004, *p* = 0.992) (Figure 2). STS time was not significantly related to absolute nor relative RFD at either early or late time interval in the REF group (absolute RFD_0–50_, *r* = 0.08, *p* = 0.797; absolute RFD_100–200_, *r* = −0.13, *p* = 0.681; relative RFD_0–50_, *r* = 0.23, *p* = 0.466; and relative RFD_100–200_, *r* = 0.004, *p* = 0.991).

### Bivariate Relationships Between Ultrasound Estimates and Force Characteristics

**FIGURE 3 |:**
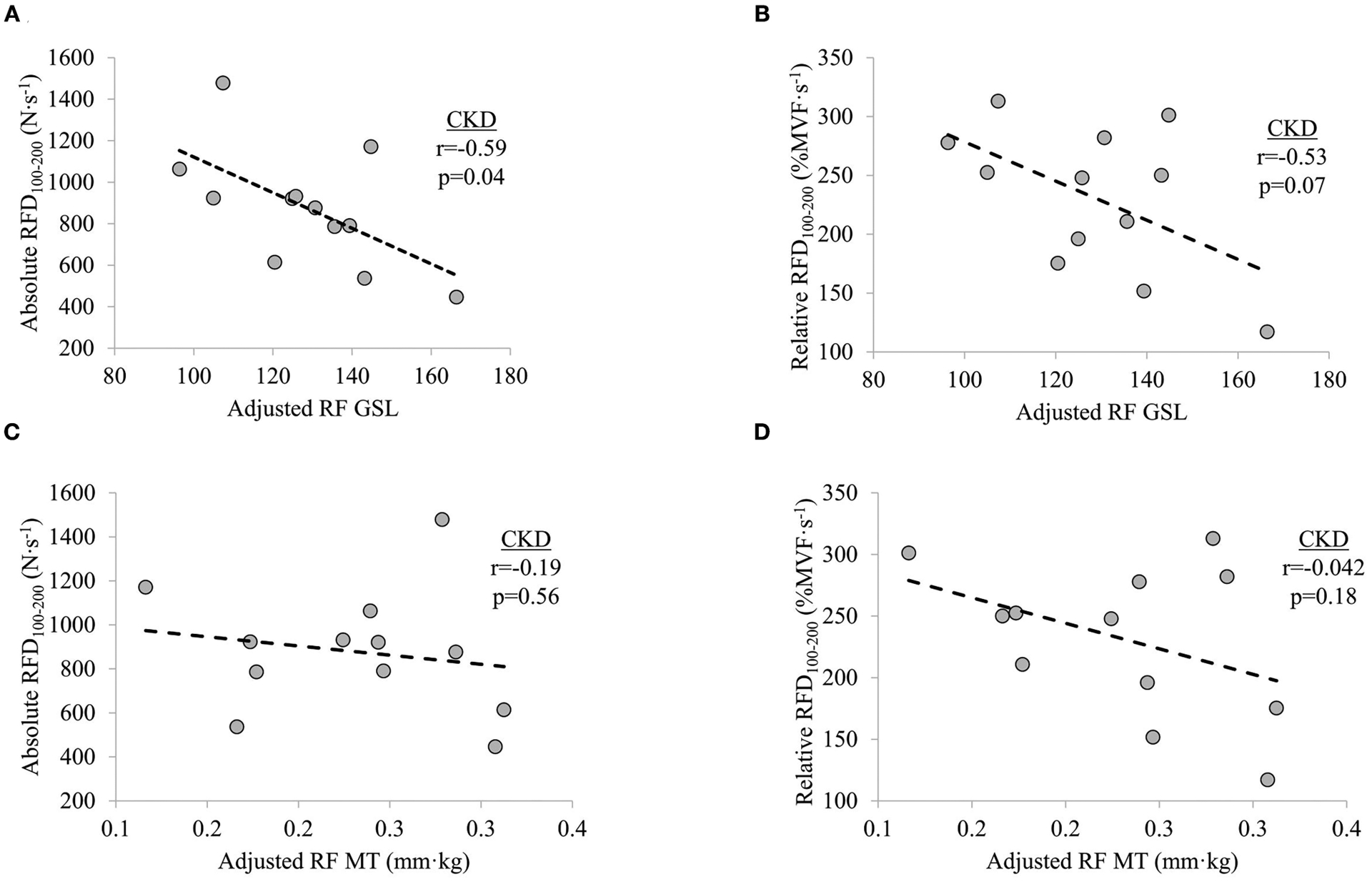
Relationships between (**A**) knee extensor absolute rate of force development (RFD) at time interval 100–200 ms (RFD_100–200_) and adjusted rectus femoris grayscale (RF GSL), (**B**) relative RFD_100–200_ and adjusted RF GSL, (**C**) absolute RFD_100–200_ and adjusted RF muscle thickness (MT), and (**D**) relative RFD_100–200_ and adjusted RF MT in patients with chronic kidney disease (CKD) stages 3b and 4 not on dialysis. Significance level set at *p* ≤ 0.05.

In participants with CKD, non-significant weak-to-moderate inverse relationships were observed between adjusted RF GSL and absolute RFD_0–50_ (*r* = −0.40, *p* = 0.200) and adjusted RF GSL and relative RFD_0–50_ (*r* = −0.24, *p* = 0.456). Significant moderate inverse relationship was observed between adjusted RF GSL and absolute RFD_100–200_ (*r* = −0.59, *p* = 0.042) while adjusted RF GSL was moderately inversely related to relative RFD_100–200_ (*r* = −0.53, *p* = 0.077), albeit non-significantly. No significant relationships were seen between adjusted RF MT and absolute RFD_0–50_ (*r* = −0.03, *p* = 0.927) or adjusted RF MT and relative RFD_0–50_ (*r* = −0.15, *p* = 0.657). Non-significant weak inverse relationship between adjusted RF MT and absolute RFD_100–200_ (*r* = −0.19, *p* = 0.560) and a non-significant moderate inverse relationship between adjusted RF MT and relative RFD_100–200_ (*r* = −0.42, *p* = 0.180) were observed, respectively (Figure 3).

## DISCUSSION

The present study examined knee extensor force characteristics and STS performance in middle-aged and older community-dwelling men with and without CKD stages 3b and 4 not on dialysis. Time in the STS task was slower, and knee extensor strength weaker, in men with CKD when compared to these measures in the REF participants. Medium and large effects were observed in relative RFD_0–50_ and relative RFD_100–200_ between groups, with relative RFD being greater in the CKD group. Significant inverse relationships between absolute and relative RFD_0–50_ and STS time were observed in the CKD group only while significant direct relationships were evident between MVF and adjusted MVF and RFD_100–200_ in the CKD and REF groups. Finally, a significant inverse relationship was found between adjusted RF GSL and absolute RFD_100–200_ in participants with CKD.

In participants with CKD, STS time was slower than in the REF group. When compared to published normative values, STS times for the REF group were consistent with those of the 70–79 age group (i.e., 12.6 s), whereas STS times of the CKD group were 23.2% slower (55). In addition to being an assessment of physical function, the STS test has also been proposed as a proxy measure of lower extremity strength and power (38, 40). The European Working Group on Sarcopenia in Older People (EWGSOP) recommends the inclusion of STS performance as a measure of muscle strength for defining and diagnosing sarcopenia (40). Using the proposed cut-off value of >15 s, 53.8% of CKD participants (*n* = 7) in the current study would be classified with low muscle strength as compared to only 16.7% of REF participants (*n* = 2) (40). Of note, data from prior investigations examining the relationship between STS performance and knee extensor strength have been inconclusive, questioning the construct validity of STS as a measure of lower extremity strength, especially when using isometric contractions (41, 54, 56–58). Therefore, other factors in addition to maximal force capacity likely influence STS performance (59).

Maximal strength plays a major role in an individual’s ability to express force rapidly (29, 30, 32, 37). We observed MVF to be directly related to late RFD in both the CKD and REF groups. It has previously been demonstrated that MVF is more strongly related to RFD during the later vs. earlier phases of isometric force generation (27–29, 32, 37). In one study, at time intervals later than 90 ms from the onset of contraction, maximal strength accounted for 52–81% of the variance in RFD, whereas at very early time intervals (<40 ms) after the onset of muscle contraction, RFD was moderately associated with twitch contractile properties, and was less related to maximal force capacity (29). In the present study, negligible-to-weak relationships were observed between absolute RFD_0–50_ and adjusted MVF in the CKD group. Conversely, a significant direct relationship was observed between absolute RFD_100–200_ and adjusted MVF, accounting for 31% of the variance. Similarly, adjusted MVF explained 48% of the variance in absolute RFD_100–200_ as compared to only 20% of the variance in absolute RFD_0–50_ in the REF group. Informed by findings from prior studies, it is inferred that factors other than MVF are primarily responsible for absolute RFD during the 0–50 ms time interval due to the minimal explained variance of RFD_0–50_ by MVF (29–33).

Declines in maximal motor unit discharge frequency and muscle quality, and alterations in muscle architecture, have all been found to contribute to reductions in RFD with aging (33, 34). Age-related differences in RFD disappeared when adjusted for peak force, suggesting that the age differences in RFD are, in part, the result of decreased maximal force (36). Although it has been demonstrated in young adults that neural activation is more strongly associated with early phases of RFD than later phases, decreases in muscle activation are thought to contribute to age-related declines in the later phases of RFD (31, 33, 34). Despite RFD being considered an important neuromuscular outcome for physical function in older adults, the corresponding evidence of the importance of RFD in patients with moderate-to-several kidney dysfunction is more limited (21–24). Reductions in renal function accelerate pathophysiological processes responsible for the neuromuscular and functional declines observed with aging (4, 60). Research on neuromuscular health in patients with kidney disease has primarily focused on muscle size, due to the increased risk for accelerated muscle loss (16, 60, 61). However, neurological alterations associated with CKD have also been described (13, 14). There is evidence to suggest alterations in neurological function at early stages of renal dysfunction, before renal failure, yet it is unclear how such alterations may influence neuromuscular force generation (13). Given the inverse relationship between early RFD and STS time in our sample of patients with stages 3b and 4 CKD, further studies are warranted to determine the specific neural and skeletal muscle factors contributing to disruptions in the early time intervals of absolute RFD.

Measures of skeletal muscle structure obtained using diagnostic ultrasonography have been found to be related to measures of force and physical function (30, 33, 46, 48, 62–69). Here, we report a significant inverse association between adjusted RF GSL and absolute RFD_100–200_ in patients with CKD. The strength of the relationships between adjusted RF GSL and measures of RFD at the time interval 0–50 ms in the present study are in agreement with that reported by Wilhelm et al. in older men (i.e., *r* = −0.40 and *r* = −0.24 vs. *r* = −0.227) (67). Similarly, echogenicity of the medial gastrocnemius was found to be more strongly related to late, as compared to early, rate of torque, consistent with the findings of the current study in knee extensor RFD (33). Despite the potential implications of RF GSL, no meaningful relationships were observed between adjusted RF MT and measures of RFD in participants with CKD. It has previously been shown that late phase rate of torque development is positively related to MT of the vastus lateralis and vastus intermedius (30), but not the RF (68). Therefore, the relationship between adjusted RF MT and late phase RFD observed in CKD participants in the present study should be interpreted within the context of architectural variability of the quadricep muscle group (70).

### Clinical Implications

Resistance exercise offers a powerful stimulus for enhancing both maximal force and RFD in older adults (71–73). To date, there has been relatively little study of the effects of resistance exercise in CKD patients not requiring dialysis, as compared with the number of investigations of aerobic and/or resistance exercise in generally healthy older adults and in patients with ESKD on dialysis. Given the relationship between maximal strength and RFD, mixed methods approaches to resistance exercise, which manipulate both exercise intensity (i.e., load) and speed of contraction, may be advantageous for addressing neuromuscular impairments and activity limitations in patients with CKD and in older adults (74). Such approaches focus on targeting specific neuromuscular characteristics (i.e., work capacity, maximal strength, power output) in a progressive and sequential manner. When working with deconditioned populations, reduced exercise intensities are required during the initial periods of an exercise regimen for safety, tolerability, and adherence. The benefits achieved during the beginning stages of an exercise regimen enable the neuromuscular system to subsequently manage greater exercise intensities. Once increases in force capacity are developed, individuals can then focus on expressing new force capabilities more rapidly. Although a recent position statement on resistance exercise recommends such approaches for older adults, further research is required to determine the effects of the manipulation of various training variables for enhancing neuromuscular capacity and physical function in patients with CKD (75).

Limitations to the present study include the following. The small sample size of older adult male veterans with and without CKD in this exploratory study precludes generalization of the current findings beyond our study sample. Additionally, the small sample may have influenced the relationships observed in the present study and therefore future large-scale studies will be necessary to confirm and extend these findings. Verbal instruction has been shown to influence the RFD achieved, making it unclear if RFD values would have been different using other methods. However, the verbal instructions provided were standardized between both groups, so that any influence of verbal instructions on RFD should have been consistent across groups. Additionally, several measures were taken to increase confidence in the analysis of RFD, including elimination of pre-tension and countermovement prior to the onset of muscle contraction, and the elimination of trials failing to demonstrate a rapid rise in the force trace immediately following the onset of contraction. Finally, further investigation is required to determine the most effective strategies for improving RFD and physical function in men and women with CKD not requiring dialysis.

## CONCLUSION

The results of the current study highlight the declines in strength and physical function that occur in older men with CKD stages 3b and 4 not requiring dialysis. Moreover, early RFD was associated with STS time in CKD while late RFD was associated MVF in both CKD and REF.

## Figures and Tables

**TABLE 1 |:** Participant demographics.

	CKD (*n* = 13)	95% CI	REF (*n* = 12)	95% CI	SMD
Age (years)	70.5 ± 6.4	[66.7,74.4]	70.2 ± 2.9	[68.3,72.0]	0.07
Weight (kg)	107.3 ± 22.8	[93.6,121.1]	96.3 ± 11.4	[89.1,103.6]	0.61
BMI (kg·m^−2^)	33.5 ± 6.3	[29.7,37.3]	31.5 ± 3.6	[29.2,33.4]	0.39
eGFR (mL/min per 1.73 m^2^)	35.1 ± 7.5	[30.6,39.6]	80.3 ± 14.8	[70.9,89.7]	−3.86
Diabetes mellitus (y/n)	9/4	*NA*	4/8	*NA*	*NA*
Hypertension (y/n)	12/1	*NA*	11/1	*NA*	*NA*
Sit-to-Stand (s)	15.9 ± 3.4	[13.6,18.2]	12.2 ± 3.7	[9.9,14.6]	1.03
MVF (*N*)	399.9 ± 95.7	[342.1,457.8]	461.4 ± 132.1	[377.4,545.3]	−0.53
Adjusted MVF (N·kg^0.67^)	17.7 ± 4.1	[15.3,20.2]	21.5 ± 5.3	[18.1,24.9]	−0.79
Absolute RFD_0–50_ (N·s^−1^)	1,848.3 ± 1,124.1	[1,169.0, 2,527.6]	1,546.2 ± 995.3	[913.8, 2,178.5]	0.29
Absolute RFD100–200 (N·s^−1^)	904.4 ± 286.3	[731.4,1077.3]	769.7 ± 420.7	[502.4, 1,037]	0.37
Relative RFD_0–50_ (%MVF·s^−1^)	503.2 ± 364.0	[305.3,701.0]	339.0 ± 233.6	[147.2,530.8]	0.54
Relative RFD_100–200_ (%MVF·s^−1^)	229.3 ± 59.3	[197.1,261.5]	162.3 ± 76.7	[118.9,205.7]	0.98
Adjusted RF MT (mm·kg)	0.2 ± 0.1	[0.2,0.3]	*N/A*	*N/A*	*N/A*
Adjusted RF GSL (unitless)	128.3 ± 19.5	[115.9,140.8]	*N/A*	*N/A*	*N/A*
RF Subcutaneous Fat (cm)	1.4 ± 0.6	[1.0,1.7]	*N/A*	*N/A*	*N/A*

CKD, participants with stages 3b and 4 chronic kidney disease; REF, reference participants without CKD; 95% CI, 95% confidence interval; SMD, standardized mean difference; eGFR, estimated glomerular filtration rate; kg, kilogram; m, meter; s, seconds; MVF, maximal voluntary isometric force; N, Newtons; N·kg^0.67^, Newtons per kilogram to two-thirds power; N·s^−1^, Newton per second; RFD, rate of force development, mm, millimeters; RF, rectus femoris; MT, muscle thickness; GSL, grayscale; y/n, yes/no; NA, not applicable.

## Data Availability

The raw data supporting the conclusions of this article will be made available by the authors, without undue reservation.

## References

[1] Gollie JM, Harris-Love MO, Patel SS, Argani S. Chronic kidney disease: considerations for monitoring skeletal muscle health and prescribing resistance exercise. Clin Kidney J. (2018) 11:822–31. doi: 10.1093/ckj/sfy054 30524717 PMC6275456

[2] Kooman JP, Dekker MJ, Usvyat LA, Kotanko P, van der Sande FM, Schalkwijk CG, Inflammation and premature aging in advanced chronic kidney disease. Am J Physiol-Ren Physiol. (2017) 313:F938–50. doi: 10.1152/ajprenal.00256.2017 28701312

[3] Painter P, Marcus RL. Assessing physical function and physical activity in patients with CKD. Clin J Am Soc Nephrol. (2013) 8:861–72. doi: 10.2215/CJN.06590712 23220421

[4] Stenvinkel P, Larsson TE. Chronic kidney disease: a clinical model of premature aging. Am J Kidney Dis. (2013) 62:339–51. doi: 10.1053/j.ajkd.2012.11.051 23357108

[5] Guideline development group, Bilo H, Coentrao L, Couchoud C, Covic A, De Sutter J, Clinical Practice Guideline on management of patients with diabetes and chronic kidney disease stage 3b or higher (eGFR <45 mL/min). Nephrol Dial Transplant. (2015) 30:ii1–ii142. doi: 10.1093/ndt/gfv100 25940656

[6] Bolton CF, Remtulla H, Toth B, Bernardi L, Lindsay RM, Maryniak O, Distinctive electrophysiological features of denervated muscle in uremic patients. J Clin Neurophysiol O Publ Am Electroencephalogr Soc. (1997) 14:539–42. doi: 10.1097/00004691-199711000-00011 9458061

[7] Campistol JM. Uremic myopathy. Kidney Int. (2002) 62:1901–13. doi: 10.1046/j.1523-1755.2002.00614.x 12371997

[8] Krishnan AV, Kiernan MC. Uremic neuropathy: Clinical features and new pathophysiological insights. Muscle Nerve. (2007) 35:273–90. doi: 10.1002/mus.20713 17195171

[9] Laaksonen S, Metsärinne K, Voipio-Pulkki L-M, Falck B. Neurophysiologic parameters and symptoms in chronic renal failure: Polyneuropathy and Hemodialysis. Muscle Nerve. (2002) 25:884–90. doi: 10.1002/mus.10159 12115978

[10] Kidney Disease: Improving Global Outcomes (KDIGO) CKD Work Group. KDIGO 2012 Clinical Practice Guideline for the Evaluation and Management of Chronic Kidney Disease. Kidney Int Suppl. (2013) 3:1–150. doi: 10.1038/kisup.2012.73

[11] Webster AC, Nagler EV, Morton RL, Masson P. Chronic Kidney Disease. Lancet. (2017) 389:1238–52. doi: 10.1016/S0140-6736(16)32064-5 27887750

[12] Painter P Roshanravan B. The association of physical activity and physical function with clinical outcomes in adults with chronic kidney disease. Curr Opin Nephrol Hypertens. (2013) 22:615–23. doi: 10.1097/MNH.0b013e328365b43a 24100215

[13] Aggarwal HK, Sood S, Jain D, Kaverappa V, Yadav S. Evaluation of spectrum of peripheral neuropathy in predialysis patients with chronic kidney disease. Ren Fail. (2013) 35:1323–9. doi: 10.3109/0886022X.2013.828261 23964701

[14] Krishnan AV, Kiernan MC. Neurological complications of chronic kidney disease. Nat Rev Neurol. (2009) 5:542–51. doi: 10.1038/nrneurol.2009.138 19724248

[15] Krishnan AV, Phoon RKS, Pussell BA, Charlesworth JA, Bostock H, Kiernan MC. Altered motor nerve excitability in end-stage kidney disease. Brain. (2005) 128:2164–74. doi: 10.1093/brain/awh558 15947058

[16] Wang XH, Mitch WE. Mechanisms of muscle wasting in chronic kidney disease. Nat Rev Nephrol. (2014) 10:504–16. doi: 10.1038/nrneph.2014.112 24981816 PMC4269363

[17] Gamboa JL, Roshanravan B, Towse T, Keller CA, Falck AM Yu C, Frontera WR, Skeletal muscle mitochondrial dysfunction is present in patients with ckd before initiation of maintenance hemodialysis. Clin J Am Soc Nephrol. (2020) 15:926–36. doi: 10.2215/CJN.10320819 32591419 PMC7341789

[18] John SG, Sigrist MK, Taal MW, McIntyre CW. Natural history of skeletal muscle mass changes in chronic kidney disease stage 4 and 5 patients: an observational study. PLoS ONE. (2013) 8:e65372. doi: 10.1371/journal.pone.0065372 23741490 PMC3669290

[19] Leikis MJ. Exercise performance falls over time in patients with chronic kidney disease despite maintenance of hemoglobin concentration. Clin J Am Soc Nephrol. (2006) 1:488–95. doi: 10.2215/CJN.01501005 17699250

[20] Padilla J, Krasno J, Da Silva M, Hsu C-Y, Frassetto L, Johansen KL, Physical functioning in patients with chronic kidney disease. J Nephrol. (2008) 21:550–9. 18651545

[21] Bento PCB, Pereira G, Ugrinowitsch C, Rodacki ALF. Peak torque and rate of torque development in elderly with and without fall history. Clin Biomech. (2010) 25:450–4. doi: 10.1016/j.clinbiomech.2010.02.002 20350773

[22] Clark DJ, Manini TM, Fielding RA, Patten C. Neuromuscular determinants of maximum walking speed in well-functioning older adults. Exp Gerontol. (2013) 48:358–63. doi: 10.1016/j.exger.2013.01.010 23376102 PMC3594593

[23] Hester GM, Ha PL, Dalton BE, VanDusseldorp TA, Olmos AA, Stratton MT, Rate of force development as a predictor of mobility in older adults. J Geriatr Phys Ther. (2021) 44:74–81. doi: 10.1519/JPT.0000000000000258 31917715

[24] Izquierdo M, Aguado X, Gonzalez R, López JL, Häkkinen K. Maximal and explosive force production capacity and balance performance in men of different ages. Eur J Appl Physiol. (1999) 79:260–7. doi: 10.1007/s004210050504 10048631

[25] Osawa Y, Studenski SA, Ferrucci L. Knee extension rate of torque development and peak torque: associations with lower extremity function: Rate of torque development and lower extremity function. J Cachexia Sarcopenia Muscle. (2018) 9:530–9. doi: 10.1002/jcsm.12285 29569834 PMC5989739

[26] Smith TM, Hester GM, Ha PL, Olmos AA, Stratton MT, VanDusseldorp TA, Sit-to-stand kinetics and correlates of performance in young and older males. Arch Gerontol Geriatr. (2020) 91:104215. doi: 10.1016/j.archger.2020.104215 32763756

[27] Maffiuletti NA, Aagaard P, Blazevich AJ, Folland J, Tillin N, Duchateau J. Rate of force development: physiological and methodological considerations. Eur J Appl Physiol. (2016) 116:1091–116. doi: 10.1007/s00421-016-3346-6 26941023 PMC4875063

[28] Rodríguez-Rosell D, Pareja-Blanco F, Aagaard P, González-Badillo JJ. Physiological and methodological aspects of rate of force development assessment in human skeletal muscle. Clin Physiol Funct Imaging. (2018) 38:743–62. doi: 10.1111/cpf.12495 29266685

[29] Andersen LL, Aagaard P. Influence of maximal muscle strength and intrinsic muscle contractile properties on contractile rate of force development. Eur J Appl Physiol. (2006) 96:46–52. doi: 10.1007/s00421-005-0070-z 16249918

[30] Cossich V, Maffiuletti NA. Early vs. late rate of torque development: Relation with maximal strength and influencing factors. J Electromyogr Kinesiol. (2020) 55:102486. doi: 10.1016/j.jelekin.2020.102486 33152680

[31] Del Vecchio A, Negro F, Holobar A, Casolo A, Folland JP, Felici F, You are as fast as your motor neurons: speed of recruitment and maximal discharge of motor neurons determine the maximal rate of force development in humans. J Physiol. (2019) 597:2445–56. doi: 10.1113/JP277396 30768687 PMC6487919

[32] Folland JP, Buckthorpe MW, Hannah R. Human capacity for explosive force production: Neural and contractile determinants: Determinants of explosive force production. Scand J Med Sci Sports. (2014) 24:894–906. doi: 10.1111/sms.12131 25754620

[33] Gerstner GR, Thompson BJ, Rosenberg JG, Sobolewski EJ, Scharville MJ. Ryan ED. Neural and Muscular Contributions to the Age-Related Reductions in Rapid Strength. Med Sci Sports Exerc. (2017) 49:1331–9. doi: 10.1249/MSS.0000000000001231 28166121

[34] Klass M, Baudry S, Duchateau J. Age-related decline in rate of torque development is accompanied by lower maximal motor unit discharge frequency during fast contractions. J Appl Physiol. (2008) 104:739–46. doi: 10.1152/japplphysiol.00550.2007 18174392

[35] Thompson BJ, Ryan ED, Herda TJ, Costa PB, Herda AA, Cramer JT. Age-related changes in the rate of muscle activation and rapid force characteristics. Age. (2014) 36:839–49. doi: 10.1007/s11357-013-9605-0 24338233 PMC4039274

[36] Thompson BJ, Ryan ED, Sobolewski EJ, Conchola EC, Cramer JT. Age related differences in maximal and rapid torque characteristics of the leg extensors and flexors in young, middle-aged and old men. Exp Gerontol. (2013) 48:277–82. doi: 10.1016/j.exger.2012.10.009 23142518

[37] Varesco G, Espeit L, Feasson L, Lapole T, Rozand V. Rate of force development and rapid muscle activation characteristics of knee extensors in very old men. Exp Gerontol. (2019) 124:110640. doi: 10.1016/j.exger.2019.110640 31252160

[38] Alcazar J, Losa-Reyna J, Rodriguez-Lopez C, Alfaro-Acha A, Rodriguez-Mañas L, Ara I, The sit-to-stand muscle power test: An easy, inexpensive and portable procedure to assess muscle power in older people. Exp Gerontol. (2018) 112:38–43. doi: 10.1016/j.exger.2018.08.006 30179662

[39] Baltasar-Fernandez I, Alcazar J, Rodriguez-Lopez C, Losa-Reyna J, Alonso-Seco M, Ara I, Sit-to-stand muscle power test: Comparison between estimated and force plate-derived mechanical power and their association with physical function in older adults. Exp Gerontol. (2021) 145:111213. doi: 10.1016/j.exger.2020.111213 33340686

[40] Cruz-Jentoft AJ, Bahat G, Bauer J, Boirie Y, Bruyère O, Cederholm T, Sarcopenia: revised European consensus on definition and diagnosis. Age Ageing. (2019) 48:16–31. doi: 10.1093/ageing/afy169 30312372 PMC6322506

[41] Takai Y, Ohta M, Akagi R, Kanehisa H, Kawakami Y, Fukunaga T. Sit-to-stand test to evaluate knee extensor muscle size and strength in the elderly: a novel approach. J Physiol Anthropol. (2009) 28:123–8. doi: 10.2114/jpa2.28.123 19483373

[42] Kollock RO, Onate JA, Van Lunen B. The reliability of portable fixed dynamometry during hip and knee strength assessments. J Athl Train. (2010) 45:349–56. doi: 10.4085/1062-6050-45.4.349 20617909 PMC2902028

[43] Jaric S, Mirkov D, Markovic G. Normalizing physical performance tests for body size: a proposal for standardization. J Strength Cond Res. (2005) 19:467. doi: 10.1519/R-15064.1 15903392

[44] Harris-Love MO, Seamon BA, Teixeira C, Ismail C. Ultrasound estimates of muscle quality in older adults: reliability and comparison of photoshop and ImageJ for the grayscale analysis of muscle echogenicity. PeerJ. (2016) 4:e1721. doi: 10.7717/peerj.1721 26925339 PMC4768702

[45] Ismail C, Zabal J, Hernandez HJ, Woletz P, Manning H, Teixeira C, DiPietro L, Blackman MR, Harris-Love MO. Diagnostic ultrasound estimates of muscle mass and muscle quality discriminate between women with and without sarcopenia. Front Physiol. (2015) 6:302. doi: 10.3389/fphys.2015.00302 26578974 PMC4625057

[46] Stock MS, Thompson BJ. Echo intensity as an indicator of skeletal muscle quality: applications, methodology, and future directions. Eur J Appl Physiol. (2020) 121:369–80. doi: 10.1007/s00421-020-04556-6 33221942

[47] Harris-Love MO, Gonzales TI, Wei Q, Ismail C, Zabal J, Woletz P, Association between muscle strength and modeling estimates of muscle tissue heterogeneity in young and old adults. J Ultrasound Med. (2018) 38:1757–68. doi: 10.1002/jum.14864 30548644 PMC9003580

[48] Stock MS, Whitson M, Burton AM, Dawson NT, Sobolewski EJ, Thompson BJ. Echo intensity versus muscle function correlations in older adults are influenced by subcutaneous fat thickness. Ultrasound Med Biol. (2018) 44:1597–605. doi: 10.1016/j.ultrasmedbio.2018.04.009 29776601

[49] Young H-J, Jenkins NT, Zhao Q, Mccully KK. Measurement of intramuscular fat by muscle echo intensity: Muscle Echo Intensity and Fat. Muscle Nerve. (2015) 52:963–71. doi: 10.1002/mus.24656 25787260 PMC4575231

[50] Austin PC. Using the standardized difference to compare the prevalence of a binary variable between two groups in observational research. Commun Stat -Simul Comput. (2009) 38:1228–34. doi: 10.1080/03610910902859574

[51] Yang D, Dalton JE. A unified approach to measuring the effect size between two groups using SAS®. SAS Glob Forum. (2012). Available online at: http://support.sas.com/resources/papers/proceedings12/335-2012.pdf (accessed January 7, 2021)

[52] Cumming G Understanding the new statistics: effect sizes, confidence intervals, and meta-analysis. New York: Routledge, Taylor & Francis Group. (2012).

[53] American College of Sports Medicine. ACSM’s Guidelines for Exercise Testing and Prescription Tenth Edition. Philadelphia, PA: Wolters Kluwer (2018).

[54] Wilkinson TJ, Xenophontos S, Gould DW, Vogt BP, Viana JL, Smith AC, Test–retest reliability, validation, and “minimal detectable change” scores for frequently reported tests of objective physical function in patients with non-dialysis chronic kidney disease. Physiother Theory Pract. (2018) 1–12. doi: 10.1080/09593985.2018.1455249 29601230

[55] Bohannon RW. Reference values for the five-repetition sit-to-stand test: a descriptive meta-analysis of data from elders. Percept Mot Skills. (2006) 103:215–22. doi: 10.2466/pms.103.1.215-222 17037663

[56] Bohannon RW. Alternatives for measuring knee extension strength of the elderly at home. Clin Rehabil. (1998) 12:434–40. doi: 10.1191/026921598673062266 9796934

[57] Ferrucci L, Guralnik JM, Buchner D, Kasper J, Lamb SE, Simonsick EM, Departures from linearity in the relationship between measures of muscular strength and physical performance of the lower extremities: the women’s health and aging study. J Gerontol A Biol Sci Med Sci. (1997) 52A:M275–M285. doi: 10.1093/gerona/52A.5.M275 9310081

[58] Harris-Love M, Benson K, Leasure E, Adams B, McIntosh V. The influence of upper and lower extremity strength on performance-based sarcopenia assessment tests. J Funct Morphol Kinesiol. (2018) 3:53. doi: 10.3390/jfmk3040053 30533549 PMC6286049

[59] Lord SR, Murray SM, Chapman K, Munro B, Tiedemann A. Sit-to-stand performance depends on sensation, speed, balance, and psychological status in addition to strength in older people. J Gerontol A Biol Sci Med Sci. (2002) 57:M539–43. doi: 10.1093/gerona/57.8.M539 12145369

[60] Stenvinkel P, Carrero JJ, von Walden F, Ikizler TA, Nader GA. Muscle wasting in end-stage renal disease promulgates premature death: established, emerging and potential novel treatment strategies. Nephrol Dial Transplant. (2016) 31:1070–7. doi: 10.1093/ndt/gfv122 25910496

[61] Fahal IH. Uraemic sarcopenia: aetiology and implications. Nephrol Dial Transplant. (2014) 29:1655–65. doi: 10.1093/ndt/gft070 23625972

[62] Fukumoto Y, Ikezoe T, Yamada Y, Tsukagoshi R, Nakamura M, Mori N, Skeletal muscle quality assessed from echo intensity is associated with muscle strength of middle-aged and elderly persons. Eur J Appl Physiol. (2012) 112:1519–25. doi: 10.1007/s00421-011-2099-5 21847576

[63] Lopez P, Wilhelm EN, Rech A, Minozzo F, Radaelli R, Pinto RS. Echo intensity independently predicts functionality in sedentary older men: Muscle Echo Intensity in Older Men. Muscle Nerve. (2017) 55:9–15. doi: 10.1002/mus.25168 27145419

[64] Mota JA, Giuliani HK, Gerstner GR, Ryan ED. The rate of velocity development associates with muscle echo intensity, but not muscle cross-sectional area in older men. Aging Clin Exp Res. (2018) 30:861–5. doi: 10.1007/s40520-017-0829-1 28936628

[65] Mota JA, Stock MS. Rectus femoris echo intensity correlates with muscle strength, but not endurance, in younger and older men. Ultrasound Med Biol. (2017) 43:1651–7. doi: 10.1016/j.ultrasmedbio.2017.04.010 28533003

[66] Rech A, Radaelli R, Goltz FR, da Rosa LHT, Schneider CD, Pinto RS. Echo intensity is negatively associated with functional capacity in older women. AGE. (2014) 36:9708. doi: 10.1007/s11357-014-9708-2 25167965 PMC4453939

[67] Wilhelm EN, Rech A, Minozzo F, Radaelli R, Botton CE, Pinto RS. Relationship between quadriceps femoris echo intensity, muscle power, and functional capacity of older men. AGE. (2014) 36:9625. doi: 10.1007/s11357-014-9625-4 24515898 PMC4082605

[68] Coratella G, Longo S, Borrelli M, Doria C, Cè E, Esposito F. Vastus intermedius muscle architecture predicts the late phase of the knee extension rate of force development in recreationally resistance-trained men. J Sci Med Sport. (2020) 23:1100–4. doi: 10.1016/j.jsams.2020.04.006 32416973

[69] Strasser EM, Draskovits T, Praschak M, Quittan M, Graf A. Association between ultrasound measurements of muscle thickness, pennation angle, echogenicity and skeletal muscle strength in the elderly. Age. (2013) 35:2377–88. doi: 10.1007/s11357-013-9517-z 23456136 PMC3824993

[70] Blazevich AJ, Gill ND, Zhou S. Intra- and intermuscular variation in human quadriceps femoris architecture assessed in vivo. J Anat. (2006) 209:289–310. doi: 10.1111/j.1469-7580.2006.00619.x 16928199 PMC2100333

[71] Borde R, Hortobágyi T, Granacher U. Dose–Response relationships of resistance training in healthy old adults: a systematic review and meta-analysis. Sports Med. (2015) 45:1693–720. doi: 10.1007/s40279-015-0385-9 26420238 PMC4656698

[72] Peterson MD, Rhea MR, Sen A, Gordon PM. Resistance exercise for muscular strength in older adults: A meta-analysis. Ageing Res Rev. (2010) 9:226–37. doi: 10.1016/j.arr.2010.03.004 20385254 PMC2892859

[73] Stewart VH, Saunders DH, Greig CA. Responsiveness of muscle size and strength to physical training in very elderly people: A systematic review: Hypertrophic ability of older muscle. Scand J Med Sci Sports. (2014) 24:e1–e10. doi: 10.1111/sms.12123 24151875

[74] Newton RU, Hakkinen K, Hakkinen A, McCormick M, Volek J, Kraemer WJ. Mixed-methods resistance training increases power and strength of young and older men. Med Sci Sports Exerc. (2002) 34:1367–75. 12165694 10.1097/00005768-200208000-00020

[75] Fragala MS, Cadore EL, Dorgo S, Izquierdo M, Kraemer WJ, Peterson MD, Resistance training for older adults: position statement from the national strength and conditioning association. J Strength Cond Res. (2019) 33:2019–52. doi: 10.1519/JSC.0000000000003230 31343601

